# Bacterial cell-free supernatants as metabolite-based biostimulants for alleviating salinity stress in plants

**DOI:** 10.3389/fpls.2026.1824871

**Published:** 2026-05-29

**Authors:** Rania Alrasheed, Judith Naamala, Sowmyalakshmi Subramanian, Donald L. Smith

**Affiliations:** Department of Plant Science, McGill University, Montreal, QC, Canada

**Keywords:** *Bacillus*, cell-free supernatant, PGPB, plant biostimulant, pseudomonas, salinity stress

## Abstract

Soil salinity severely limits global crop productivity, affecting approximately 20% of irrigated land under changing climate conditions. It impairs water uptake, disrupts nutrient balance, and induces oxidative stress, leading to significant yield losses, particularly in salt-sensitive crops. This review evaluates the potential of metabolite-rich bacterial cell-free supernatants (CFS) derived from plant growth-promoting bacteria (PGPB) as biostimulants for mitigating salinity stress in crops including soybean, canola, and wheat. CFS contain diverse bioactive compounds, including phytohormones, osmolytes, antioxidants, microbe-to-plant signal compounds and nutrient-solubilizing agents, which enhance plant performance through mechanisms such as modulation of hormonal signaling, activation of antioxidant defenses, and regulation of ion homeostasis. Compared to living-cell microbial inoculants, CFS offer advantages in stability and regulatory acceptance. However, their practical application is constrained by variability in composition, lack of standardized protocols, and limited field validation. Addressing these challenges through targeted research and field studies will be critical to enable the adoption of CFS-based strategies for climate-resilient agriculture.

## Graphical Abstract

**Figure:**
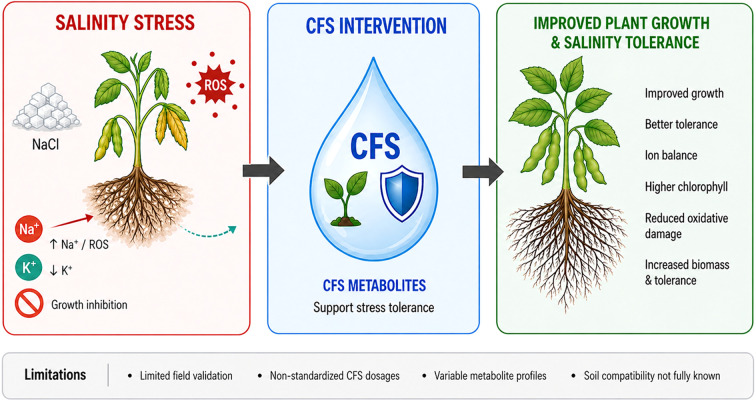
Conceptual overview of salinity−induced ion imbalance and oxidative stress in plants, and the mitigating effects of metabolite−rich bacterial cell−free supernatants (CFSs) that enhance ion homeostasis, reduce oxidative damage, and support improved growth under saline conditions.

## 1 Introduction

### 1.1 Soil salinity in agriculture

Soil salinity is a global abiotic stressor especially in arid/semi-arid regions, resulting from the accumulation of excessive soluble salts containing ions such as Na^+^, Cl⁻, S_4_²⁻, and CO_3_²⁻ in soil/irrigation water, which severely impair crop growth and yield, hence affecting agricultural productivity. It affects over 1.4 B ha of land globally, with projections indicating a threefold increase by 2100 due to climate change, unsustainable irrigation strategies, and rising sea levels (FAO, 2024). Soils are generally classified as saline when the electrical conductivity (ECe) of the saturated paste extract exceeds 4 dS/m at 25 °C (approximately 40 mM NaCl), a level that disrupts growth in many crop species (Richards, 1954; Shrivastava and Kumar, 2015; Zörb et al., 2019; Lindberg and Premkumar, 2024). Salinity stress can be broadly classified into primary (natural) salinity and secondary (anthropogenic) salinity. Primary salinity results from rock weathering, upward movement of saline groundwater, and marine water intrusion, particularly in low-rainfall regions where limited precipitation prevents salt leaching (Shrivastava and Kumar, 2015; Corwin, 2021). Secondary salinity is caused by human activities such as poor irrigation practices, overuse of saline irrigation water, inadequate drainage, excessive fertilizer application, and deforestation, which collectively increase the salt burden in soils (Chele et al., 2021; Corwin, 2021; Ullah et al., 2021). Globally, salinity affects about one-third of irrigated land roughly 20% of cultivated land, which together contribute a disproportionately large share of the world’s food production lands, and these areas are projected to expand further under current management and developing climate trends (Chele et al., 2021; Alotaibi, 2023). Salinity not only diminishes crop yields but also causes substantial economic losses, with recent global assessments warning of escalating costs as more croplands become salt-affected (Chele et al., 2021; FAO, 2024).

Yield losses of approximately 30-50% in staples such as soybean and canola were reported (Ilangumaran and Smith, 2017; Ilyas et al., 2024). Research also indicates that soybean yields can decline by over 20% for each increase of 1 dS m^-1^ in ECe above the 5 dS m^-1^ threshold (Li et al., 2024a, b; Stewart and Lee, 2024). Additionally, canola yields can drop by more than 50% when salinity levels exceed 10 dS m^-1^, particularly on poor-quality saline soils. Wheat also suffers significantly, with reported yield losses ranging from 30 to 70% when ECe levels surpass 8 dS m^-1^, contributing to markedly reduced biomass production (MaChado and Serralheiro, 2017). These losses are particularly significant in high-latitude semi-arid regions, such as the Canadian Prairies, where increasing soil salinity intersects with short growing seasons to threaten the stability of oilseed and pulse production (Government of Canada, 2023; Government of Canada, 2020). These findings highlight the urgent need for effective management strategies to mitigate salinity-induced stress on crops (Aiad et al., 2021). Regional economics also underscore this urgency. For example, California’s Great Central Valley projects estimates annual losses of $191–231 million by 2030 due to declining agricultural productivity driven by soil salinity (Hameed et al., 2024).

**Figure 1:**
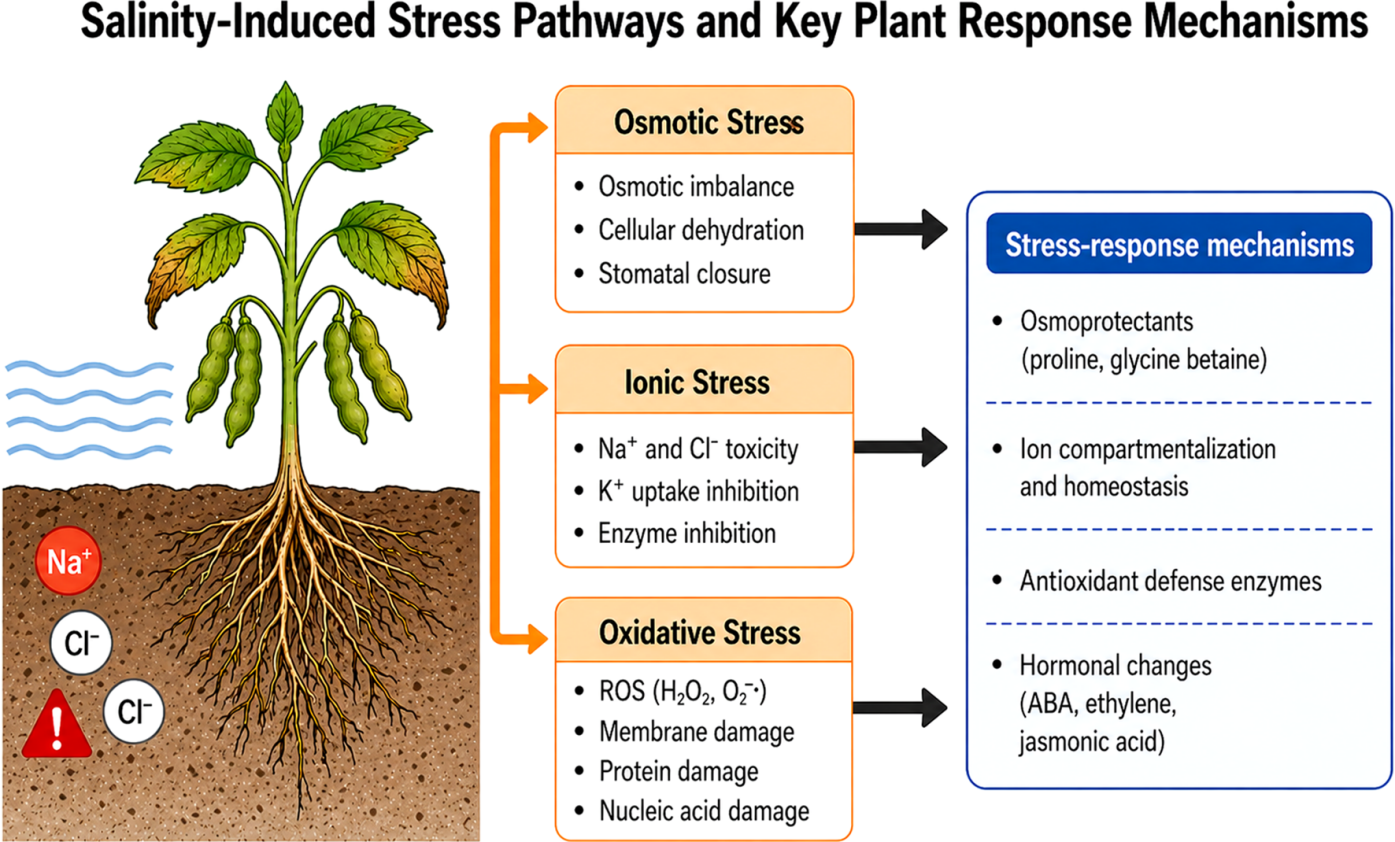
Major physiological phases of plant responses to NaCl salinity. The initial osmotic phase reduces water uptake and leaf expansion, followed by ionic stress caused by Na^+^ and Cl⁻ accumulation and disruption of K^+^ homeostasis. Prolonged exposure results in oxidative stress characterized by excessive reactive oxygen species, membrane damage, and reduced photosynthetic efficiency.

The impact of salinity stress on crop productivity stems from three interconnected physiological stress phases, namely, osmotic stress, ionic stress, and oxidative stress, which collectively impair key metabolic pathways, including the carbon fixation (photosynthesis) system activity, undermining crop productivity and quality under saline conditions. Osmotic stress is triggered by the exposure of plants to salt stress, where high external salt concentrations reduce soil water potential, which makes it difficult for plant roots to absorb water and may cause water flow from plant cells. Consequently, plant cells lose turgor pressure, causing symptoms such as leaf wilting and stunted growth. Plants respond by closing their stomata to conserve water, which restricts CO_2_ uptake and slows photosynthesis and leaf expansion (Balasubramaniam et al., 2023). Ionic stress occurs when plant salt exposure persists, resulting in the accumulation of ions such as Na^+^ and Cl⁻ ions in plant tissues, replacing essential ions such as K^+^ and Ca²^+^. This disrupts enzyme functions, protein synthesis, and membrane integrity, leading to growth inhibition and cellular damage (Fu and Yang, 2023). Prolonged exposure to salinity also causes oxidative stress which prompts the overproduction of reactive oxygen species (ROS) such as hydrogen peroxide (H_2_O_2_) and superoxide (O_2_⁻). If not neutralized by antioxidant systems, these ROS cause lipid peroxidation, protein and DNA damage, and ultimately programmed cell death (Nefissi Ouertani et al., 2021). The key components of osmotic, ionic, and oxidative stress and their associated defense pathways are summarized in **Figure 1**.

### 1.2 Plant response to salinity stress

**Figure 2:**
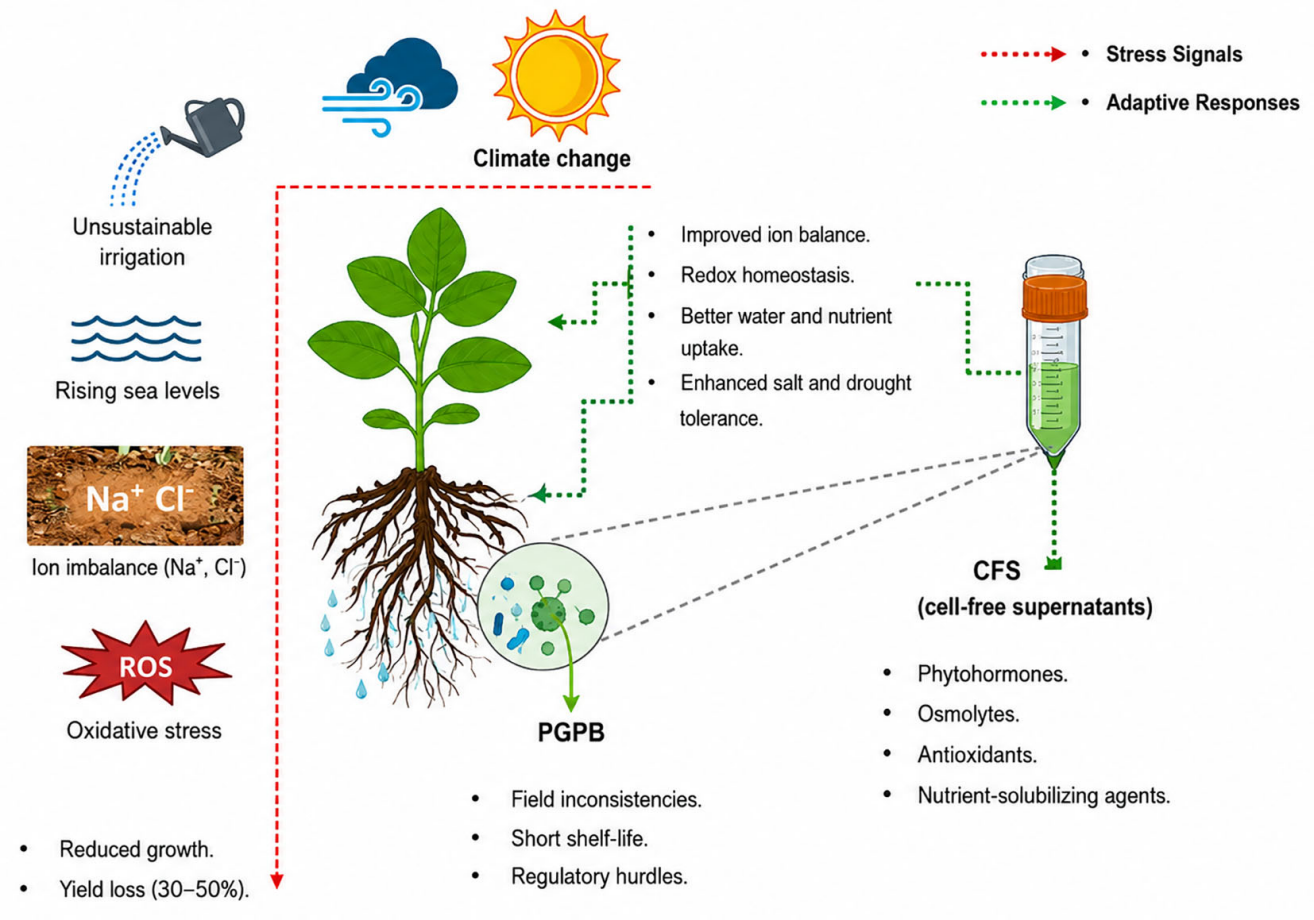
Conceptual overview of bacterial cell-free supernatants as metabolite-based biostimulants for improving plant growth and salinity stress tolerance. Salinity induces osmotic, ionic, and oxidative stress, leading to disrupted ion homeostasis, reactive oxygen species accumulation, and growth inhibition. PGPB and their CFSs mitigate these effects through hormonal modulation, antioxidant activation, improved ion balance, and osmotic adjustment.

To survive salinity stress, plants activate multiple stress-response and defense mechanisms aimed at maintaining cellular homeostasis and mitigating cell damage. One primary response is the accumulation of osmoprotectants such as proline and glycine betaine, which help maintain osmotic balance, preserve cellular integrity, and limit damage under saline conditions (Stewart and Lee, 1974; Rhodes and Hanson, 1993; Hayat et al., 2012; Roy et al., 2021; Singh et al., 2022b). Plants also compartmentalize excess sodium ions into vacuoles, thereby reducing their interference with cytosolic enzymes and metabolic processes (Greenway and Munns, 1980; Munns and Tester, 2008; Roy et al., 2021). In parallel, antioxidant enzymes such as superoxide dismutase (SOD), catalase (CAT), and ascorbate peroxidase (APX) are activated to detoxify reactive oxygen species generated by salinity, protecting key physiological and biochemical functions (Apel and Hirt, 2004; Yu et al., 2020). Abscisic acid ABA, together with other phytohormones such as jasmonic acid and ethylene, play a central role in coordinating these defense responses by modulating the expression of stress−responsive genes and adjusting growth and metabolism under saline conditions (Munns and Tester, 2008; Van de Mortel et al., 2012; Torun et al., 2022). Despite these intrinsic defenses, salt−sensitive genotypes often show limited resilience (Roy et al., 2021). This, coupled with the global socio-economic repercussions of salinity and associated threats to food security emphasize the need for external strategies based on multidisciplinary interventions, adaptation strategies and approaches which integrate agronomy, soil science, and economic assessments to develop holistic solutions to mediate salinization in agriculture. These may include implementing sustainable land management practices, investing in salinity-tolerant crop varieties, and the use of biostimulants. However, using conventional approaches such as breeding for salt−tolerant cultivars and chemical amendments, are constrained by genetic complexity, environmental variability, costs, and ecological trade−offs (Collard and Mackill, 2008; Moose and Mumm, 2008; Gaba et al., 2021; Rehman et al., 2025). Plant growth-promoting bacteria (PGPB) offer a sustainable and environmentally friendly alternative, but deployment in saline soils is hampered by inconsistent field performance, limited shelf life, and regulatory hurdles (El Moatassem et al., 2025). Their derivatives such as cell-free supernatants (CFS) and purified microbe-to-plant signal compounds could be more stable under field conditions with similar benefits to the plant. This review therefore details the role of bacterial cell-free supernatant in alleviating soil salinity as summarized in **Figure 2**. A brief background on PGPB and their modes of action is also given.

### 1.3 Literature search strategy

Relevant studies were identified using Web of Science, Scopus, and Google Scholar, covering publications from 2008 to 2026. Search terms included combinations of “cell-free supernatant”, “culture filtrate”, “bacterial metabolites”, “*Bacillus*”, “*Pseudomonas*”, “salt stress”, and “salinity”. Studies were included if they reported physiological or biochemical responses of plants to PGPB-derived CFSs or purified metabolites under defined salinity conditions. Studies focusing exclusively on live microbial inoculants without separate metabolite or CFS treatments, or lacking salinity controls, were excluded from primary analysis. The initial pool of articles was screened based on title, abstract, and full text to ensure relevance. Reference lists of key studies and prior reviews were also examined to identify additional relevant literature. This review does not follow a formal systematic review protocol but provides a structured synthesis of current evidence.

## 2 Biostimulants for salinity stress alleviation

### 2.1 Plant growth-promoting bacteria

Recent research emphasizes the importance of the rhizosphere microbiome in conferring salinity tolerance. Under salt stress, root exudation patterns change, reshaping the composition and activity of rhizosphere microbial communities (Wu et al., 2018; Sunita et al., 2020). Beneficial microbes can enhance plant resilience by secreting metabolites that prime plant defense systems, improve nutrient acquisition, and suppress pathogens (Kumar A. et al., 2020; Gupta et al., 2022). Recent advances further highlight the importance of microbial metabolites in mediating plant stress tolerance. For example, Zholdasbek et al. (2026) demonstrated that metabolite-rich extracts from halotolerant rhizobacteria significantly enhanced osmotic adjustment and ROS detoxification in wheat under high salinity, largely through modulation of proline metabolism and antioxidant enzyme activation. Similarly, Rakhmatova et al. (2026) reported that microbial metabolite fractions containing organic acids, phenolics, and low−molecular−weight peptides improved ion homeostasis and reduced Na^+^ accumulation in salt−stressed barley. These studies reinforce the growing consensus that microbial metabolites, independent of live colonization, play a central role in shaping plant adaptive responses to salinity. Certain PGPB produce secondary metabolites that simultaneously promote growth and increase stress tolerance, illustrating the potential of exploiting these microbe–plant interactions for salinity management (Gupta et al., 2022; Acharya et al., 2024).

#### 2.1.1 Overview of *Bacillus* and *Pseudomonas* as plant growth promoting bacteria

**Figure 3:**
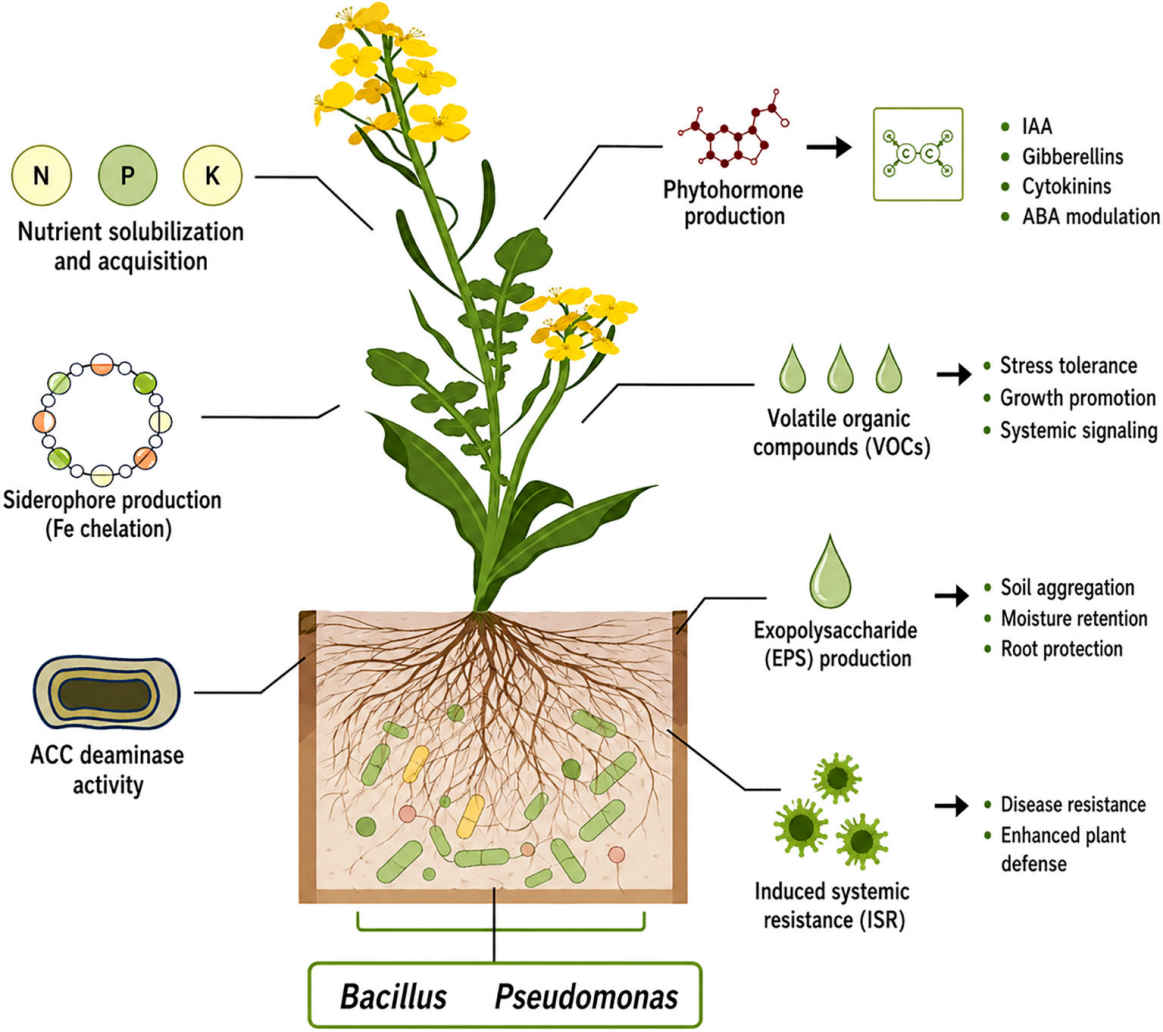
Plant growth−promoting mechanisms of root−associated *Bacillus* and *Pseudomonas*. These bacteria colonize the rhizosphere and endosphere, mobilize nutrients, produce phytohormones, express ACC deaminase activity, and induce systemic resistance or tolerance. They also enhance root architecture and antioxidant capacity, supporting plant performance under salinity stress.

*Bacillus* and *Pseudomonas* are among the leading non-symbiotic PGPB, known for their ability to enhance plant performance under abiotic stresses such as salinity and drought (Backer et al., 2018; Ajijah et al., 2023). They commonly inhabit the rhizosphere and, in some cases, the endosphere supporting plant health through diverse direct and indirect mechanisms. *Bacillus* spp. are notable for endospore formation and robust formulation potential, whereas *Pseudomonas* spp. are strong rhizosphere colonizers with diverse secondary metabolisms. Inoculation of plants with either or a combination of the two species can complement plant defenses against stress while maintaining or even enhancing plant components such as leaf area, photosynthetic rate, root-to-shoot ratios, and deeper and more branched root systems that enhance water uptake thereby conferring tolerance to both drought and salinity (Abdelkefi et al., 2024). To enhance plant health and productivity, *Bacillus* and *Pseudomonas spp* employ mechanisms such as nutrient mobilization (P and K solubilization, biological N_2_ fixation, siderophore−mediated Fe acquisition), production of phytohormones (auxins, gibberellins, cytokinins), ACC deaminase activity, induction of systemic resistance/tolerance (ISR/IST), and enhancement of osmoprotection and antioxidant regulation (Olanrewaju et al., 2017; Mohammed et al., 2020). An overview of these mechanisms is presented in **Figure 3**. The mechanisms are mediated by secreted metabolites, which accumulate in the culture medium during microbial population growth. This metabolite−driven action provides the mechanistic foundation for the bioactivity of CFSs derived from these bacteria. Several studies have demonstrated the effectiveness of the two strains in alleviating salt stress, as shown in **Table 1**. For example, *Bacillus toyonensis* COPE52 improved tomato biomass and altered membrane composition under 100 mM NaCl, while *Pseudomonas koreensis* MU2 enhanced soybean salt tolerance by optimizing ion homeostasis and activating gibberellic acid and antioxidant responses (Adhikari et al., 2020).

**Table 1:** Representative *Bacillus* and *Pseudomonas* strains and or components, their host crops, observed plant benefits, and underlying mechanisms.

Evidence type	Bacterial strain	Host plant	Salinity context	Observed benefit	Key mechanisms	Limitations/research gaps	Reference
Bacterial cells	*Bacillus toyonensis* COPE52	Tomato	100 mM NaCl	Increased biomass, chlorophyll	IAA production, membrane modulation	No CFS validation; metabolite effects inferred	Fan and Smith, 2022; Abdelkefi et al., 2024
Bacterial cells	*Bacillus subtilis*	Canola	Salinity	Enhanced root growth	ACC deaminase, siderophores	No metabolite profiling; no dose–response	Desoky et al., 2020
Bacterial cells	*Bacillus amyloliquefaciens* EB2003A	Soybean	NaCl stress	Improved germination, radicle growth	Hormonal regulation, antioxidants	CFS vs metabolites not separated	Naamala et al., 2022
Bacterial cells	*Pseudomonas koreensis* MU2	Soybean	Salinity	Reduced Na^+^ uptake, improved growth	GA production, ion homeostasis, antioxidants	No direct CFS application; greenhouse-based	Adhikari et al., 2020
Bacterial cells	*Pseudomonas fluorescens*	Wheat	Salinity	Improved growth and nutrient uptake	ACC deaminase, N metabolism	Field validation lacking	Acharya et al., 2020
CFS	*Bacillus amyloliquefaciens* CFS	Soybean, corn	50–75 mM NaCl	Improved germination and vigor	Extracellular metabolites (unresolved)	Active compounds not chemically identified	Naamala et al., 2022
CFS	*Bacillus* spp. CFS	Corn	100–150 mM NaCl	Enhanced seed vigor index	Metabolite-mediated effects	Composition not characterized	Yaghoubian et al., 2022
CFS	*Devosia* sp. SL43 CFS	Canola, soybean	Salinity	Improved seed germination (priming)	CFS-mediated stimulation	Early-stage evidence only	Shah et al., 2022
CFS	*Nocardioides* sp. BCFE	Wheat	Salinity	Improved growth and antioxidant activity	IAA, salicylate detected	Not *Bacillus*/*Pseudomonas*; crude extract	Meena et al., 2020
Metabolite extract	Halotolerant rhizobacteria (mixed metabolites)	Wheat	100–150 mM NaCl	Improved osmotic adjustment, reduced MDA	Proline metabolism, antioxidant activation	Greenhouse only; metabolite composition partially resolved	Zholdasbek et al., 2026
Metabolite fraction	Rhizobacterial low-MW metabolites	Barley	Salinity	Improved K^+^/Na^+^ ratio, reduced Na^+^ uptake	Organic acids, phenolics, peptides	No CFS comparison; limited field validation	Rakhmatova et al., 2026
Purified compound	VOCs from *Pseudomonas simiae*	Soybean	Salinity	Reduced Na^+^, increased growth and proline	Volatile signaling pathways	Mixture not fully resolved	Vaishnav et al., 2015; 2016
Purified compound	IAA, salicylate (CFS-associated)	Wheat	Salinity	Enhanced antioxidant defense	Hormonal signaling	Not tested as isolated compounds	Meena et al., 2020

Despite their well−documented mechanisms, the performance of live PGPB inoculants is often inconsistent under field conditions because bacterial survival and activity are highly sensitive to soil salinity, temperature fluctuations, moisture variability, competition from micro-flora already present and pH. Moreover, farmers have limited control over the quantity and composition of metabolites released by live bacteria in the rhizosphere, which contributes to variable outcomes and poor reproducibility across environments. These limitations have driven growing interest in microbial derivatives, particularly CFSs and purified metabolites, which offer greater stability, standardized dosages, and reduced dependence on soil conditions. **Table 1** summarizes representative studies evaluating both live inoculants and their metabolite−based derivatives under salinity stress.

As summarized in **Table 1**, both live *Bacillus* and *Pseudomonas spp* inoculants and their metabolite−based derivatives can enhance plant performance under salinity stress, but the evidence also highlights several recurring limitations. Many studies rely on greenhouse conditions, lack standardized dosages, or infer metabolite activity from plant responses without direct chemical profiling or understanding of mode of action. In addition, essential controls, such as uninoculated culture medium processed in parallel, are often missing, making it difficult to attribute effects specifically to bacterial metabolites. These gaps underscore the need for approaches that offer greater stability, reproducibility, and mechanistic clarity, which has led to increasing interest in CFSs as a more controlled alternative to inoculants based on living microbial cells.

### 2.2 Cell-free supernatants

In this review, we use the term “cell−free supernatant” or “CFS” to refer specifically to sterile microbial culture filtrates without any microbial cells, derived from PGPB grown in defined media, unless otherwise specified.

Cell-free supernatants are broth cultures prepared by culturing beneficial bacterial strains in suitable growth media, under controlled conditions, followed by centrifugation and filtration to remove viable cells (Pellegrini et al., 2020; Mani‐López et al., 2021; Subramanian et al., 2021; Msimbira et al., 2022; Qadi et al., 2023; Trinh et al., 2025). They contain a complex mixture of bioactive metabolites such as phytohormones, organic acids, siderophores, peptides, and antioxidants (Morcillo et al., 2022; Qadi et al., 2023; Alori et al., 2025). The metabolites are mostly secondary metabolites synthesized by the microbe in response to environmental stimuli. Owing to their bioactivity, CFSs can be used as biostimulants, using deployment methods such as seed priming, soil drenching and foliar spray, under stressed and non-stressed conditions (Shah et al., 2022; Naamala et al., 2022). They could circumvent several limitations associated with live microbial inoculants, such as inconsistent colonization and stress sensitivity (Wang et al., 2022). They can stimulate plant growth, improve nutrient acquisition and enhance tolerance to abiotic stresses, including salinity stress (Pellegrini et al., 2020; Morcillo et al., 2022; Naamala et al., 2023; Alori et al., 2025). The quality of CFSs as biostimulants is dependent on the quality and quantity of bioactive compounds produced by the microbe, which is also dependent on factors such as the growth media (carbon and nitrogen sources, mineral content) and culture conditions (pH, temperature, aeration, incubation duration, etc.) which modulate both the metabolic footprint and the resulting biostimulant activity (Msimbira et al., 2022; Qadi et al., 2023; Zanetta et al., 2023). For example, lactic acid bacteria grown at various initial pH values or in distinct media formulations produce CFSs with markedly different antibacterial and antioxidant properties and distinct metabolite profiles (Pellegrini et al., 2020; Abdelkefi et al., 2024). Likewise, altering culture pH prior to CFS preparation can change the effects on plant seed germination and seedling growth, indicating that stress−like culture conditions can shift the spectrum of secreted compounds (Trinh et al., 2025; Mani-López et al., 2022). Consequently, standardized reporting of culture conditions (medium formulation, incubation time, cell density at harvest, and post-processing steps) is essential to enable reproducibility and meaningful comparison across CFS studies. The careful selection and optimization of growth media and environmental conditions are therefore critical for maximizing the desired functional activities of CFSs, whether the goal is to stimulate plant growth, enhance stress resilience, or target specific pathogens. Despite promising results in controlled environments, CFS applications remain underexplored in field conditions, especially for salt−sensitive crops (Moradi et al., 2019; Pellegrini et al., 2020; Naamala et al., 2023; Alori et al., 2025). Most studies focus on model systems, leaving critical gaps, including dosage standardization, cost-effective production, and soil-compatibility testing. Moreover, many reports infer the presence or activity of metabolites such as auxins, gibberellins, or osmolytes from plant phenotypes rather than direct chemical profiling and frequently do not include essential controls such as uninoculated culture medium processed in parallel. To address these limitations, this review is presented as a narrative review supported by a structured literature search. By linking molecular insights with agronomic performance, we position CFS−based technologies as emerging tools for building salt−resilient cropping systems.

To further address the limited chemical characterization of CFSs in the literature, we explicitly distinguish between studies that directly quantify metabolites and those that infer metabolite activity from plant physiological responses. While CFSs are frequently described as rich sources of phytohormones, osmolytes, and antioxidants, the level of empirical support for these claims varies considerably across studies. A key methodological divergence exists between studies employing analytical platforms such as HPLC, LC-MS/MS, or GC-MS for direct metabolite profiling and those relying solely on phenotypic proxies, such as changes in root architecture, chlorophyll content, or ion homeostasis. **Table 2** summarizes this distinction by categorizing studies according to the nature of the evidence provided. Notably, reliance on phenotypic inference alone limits mechanistic interpretation and may lead to overestimation of specific metabolite functions.

**Table 2:** Methodological evidence for CFS bioactive compounds: direct profiling vs. phenotypic inference.

Evidence category	Studies with direct metabolite profiling	Methods used	Key compounds detected	Studies with phenotypic inference only	Phenotypic indicators
Phytohormones	Subramanian et al., 2021; Fan and Smith, 2022; Abdelkefi et al., 2024	HPLC, LC-MS	IAA, GAs, ABA, spermidine	Kasotia et al., 2015; Fan, 2017; Adhikari et al., 2020	Root growth, ion homeostasis, GA responses
Antioxidants and Osmolytes	Alori et al., 2025; Goszcz et al., 2025, and Trinh et al., 2025	GC-MS, spectrophotometry	Organic acids, proline, phenolics	Kumar et al., 2020; Shah et al., 2022; El-Gendi et al., 2022; Xie et al., 2022	SOD/CAT/APX ↑, MDA ↓, proline ↑
Siderophores and Nutrients	Subramanian, 2013; Wang et al., 2022	CAS assay, colorimetric	Siderophores, P solubilizers	Hashem et al., 2019; Briard et al., 2015	Nutrient uptake, growth promotion
ACC Deaminase and Others	Tyśkiewicz et al., 2022	Enzyme assays	ACC deaminase activity	Yasmin et al., 2020	Ethylene ↓, senescence ↓
Mixed Evidence	Peng et al., 2023; Dhar et al., 2024	HPLC + physiology	IAA linked to phenotypes	AbdAllah et al., 2018	Biomass ↑, chlorophyll ↑

In the context of this review, direct profiling refers to the analytical quantification (e.g., HPLC, GC-MS) of metabolites within the CFS before application. In contrast, phenotypic inference denotes the attribution of specific metabolite functions based solely on observed plant physiological shifts. As illustrated in **Table 2**, approximately 47% of the cited studies provide direct chemical evidence, while the remainder relies on physiological proxies. This distinction highlights a critical research gap and underscores the need for more comprehensive metabolite profiling to transition from putative to confirmed mechanistic models of CFS-mediated salt tolerance. Future work should therefore prioritize coupling plant physiological assays with untargeted and targeted metabolomics of CFSs to identify causal metabolite-trait relationships. ↑ indicates increase or upregulation; ↓ indicates decrease or downregulation. Abbreviations: SOD, superoxide dismutase; CAT, catalase; APX, ascorbate peroxidase; MDA, malondialdehyde; ACC, 1-aminocyclopropane-1-carboxylic acid; GSH, glutathione (reduced); GSSG, glutathione (oxidised).

#### 2.2.1 Mechanisms of CFS-mediated salinity stress alleviation

**Figure 4:**
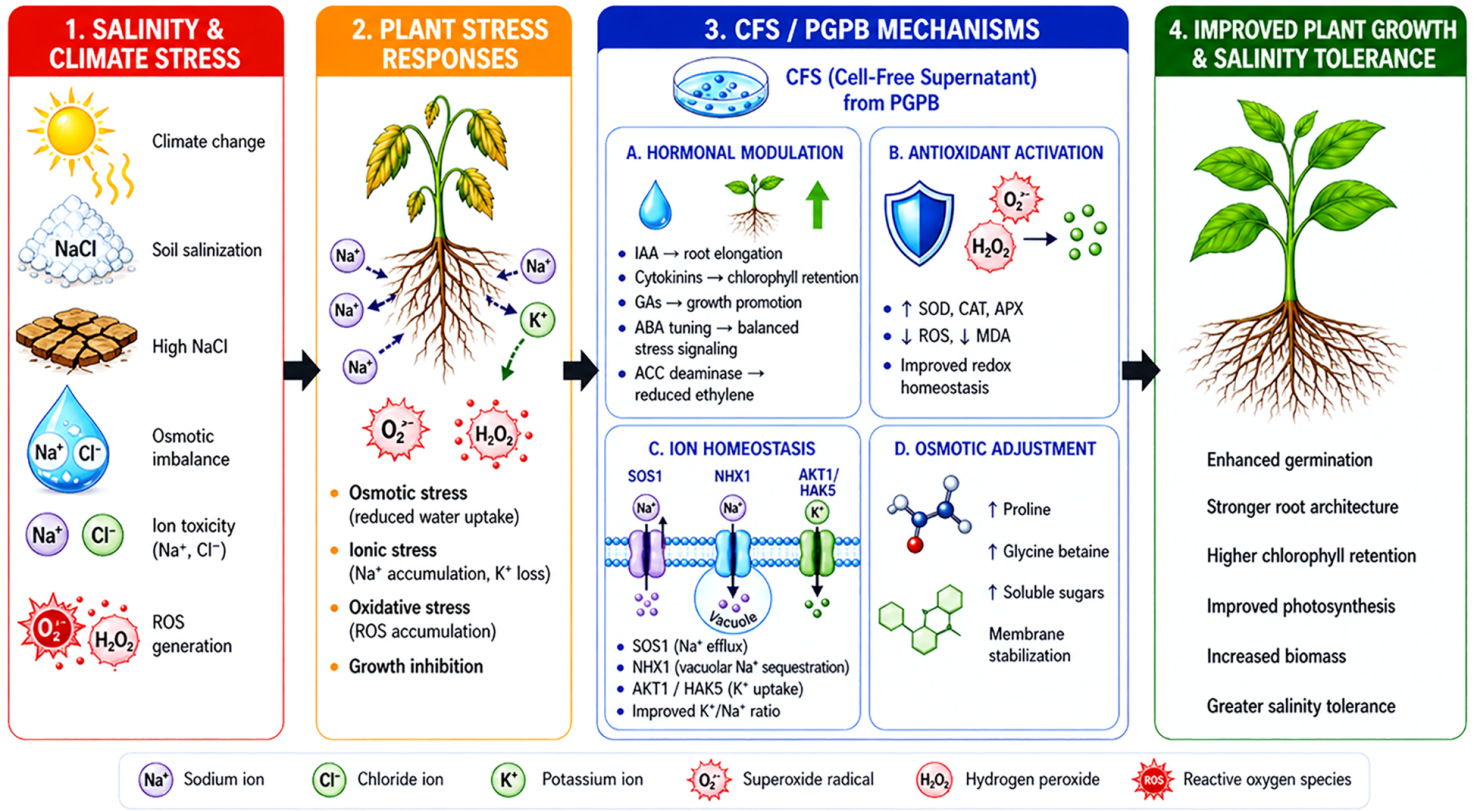
Mechanisms by which cell−free supernatants (CFSs) from plant growth−promoting bacteria alleviate salinity stress in plants. CFSs mitigate osmotic, ionic, and oxidative stress through hormonal modulation, antioxidant activation, ion homeostasis, and osmotic adjustment, collectively improving germination, root development, chlorophyll retention, photosynthesis, biomass, and salinity tolerance.

Bacterial CFSs often alleviate salinity stress in plants through modulation of hormonal signaling pathways. Many CFSs contain microbe-derived phytohormones such as indole-3-acetic acid (IAA), cytokinins, and gibberellins (GAs), which activate key hormone cascades that promote plant growth and stress adaptation. In addition to phytohormones, CFSs are often rich in osmoprotectants such as proline, organic acids, and antioxidants that help mitigate oxidative damage under salt stress conditions, while siderophores facilitate nutrient mobilization (Goszcz et al., 2025). The concentration and composition of these metabolites vary with bacterial strain and growth conditions, influencing their efficacy across different plant systems. For example, CFSs from *Devosia* sp. (Tiwari et al., 2017) have demonstrated significant benefits in enhancing soybean seed germination under salinity stress conditions, largely attributed to these bioactive compounds. Similarly, *Bacillus amyloliquefaciens* strains produce spermidine, auxins, abscisic acid, and gibberellic acid, which contribute to salt stress tolerance by maintaining ion homeostasis and boosting antioxidant enzyme activities (Naamala et al., 2022). Recent work with *Bacillus* and *Curtobacterium* endophytes further supports this multi-hormonal mode of action, linking bacterial ABA, JA, IAA, GAs and ACC deaminase with enhanced growth, reduced ethylene and improved oxidative stress tolerance under salinity (Khan et al., 2019; Kumar R. et al., 2020). These interacting hormonal, osmoprotective, and antioxidant pathways collectively contribute to CFS−mediated salinity tolerance, as summarized in **Figure 4**.

##### 2.2.1.1 Microbe-to-plant signaling

Beyond phytohormones and osmoprotectants, several CFS−derived metabolites act as microbe−to−plant signaling molecules that trigger plant stress−response pathways at very low concentrations. These include well−characterized elicitors such as lipo−chitooligosaccharides and peptide−based signals, which modulate ion homeostasis, antioxidant activity, and growth-stress trade−offs through receptor−mediated pathways (Subramanian and Smith, 2015; Gough and Cullimore, 2011, Cullimore et al., 2023). Microbial volatile organic compounds (VOCs) also contribute to salinity tolerance by functioning as airborne cues that influence ROS balance and ion transport (Fincheir et al., 2021; Fincheira and Quiroz, 2018; Vaishnav and Chowdhury, 2023).

Collectively, these signaling molecules complement the hormonal and metabolic mechanisms described above, highlighting that CFSs can fine−tune plant stress responses not only through metabolite supply but also through elicitor−driven activation of plant signaling networks.

##### 2.2.1.2 Hormonal signaling modulation

Indole-3-acetic acid (IAA) and IAA-like compounds present in CFSs can influence auxin signaling pathways in plants. Auxin perception through TIR1/AFB F-box receptors promotes degradation of AUX/IAA repressors and activation of auxin response factors (ARFs), leading to the expression of genes associated with lateral root formation (e.g., LAX3, GH3, and PIN family genes). This auxin-regulated transcriptional response enhances root elongation and root surface area, thereby improving water and nutrient uptake under saline conditions (Pellegrini et al., 2020). The improved root architecture frequently observed in CFS-treated plants under salt stress is consistent with activation of auxin-responsive growth pathways. Similar patterns of root−length enhancement, under 100–125 mM NaCl, are observed in soybean treated with *Devosia* sp. SL43−derived cell−free supernatant and associated flavonoid fractions, where root−length increases of up to ≈140–160% coincide with improved seed vigor and stress−tolerant metabolite profiles, supporting the interpretation that CFSs modulate auxin−related growth pathways under salinity (Shah et al., 2022). Cytokinins detected in CFSs may also contribute to stress mitigation by influencing cytokinin signaling networks. Cytokinin perception via CRE1/AHK histidine kinase receptors initiates a phosphorelay cascade that activates type-B response regulators (ARRs), which promote chloroplast maintenance, delay senescence, and sustain cell division. These processes are particularly important under salinity stress, where premature senescence, including chlorophyll degradation, commonly limit shoot productivity (Arkhipova et al., 2007). Physiological responses observed following CFS application, such as enhanced chlorophyll retention and shoot growth, align with cytokinin-mediated stress tolerance mechanisms. Consistently, biostimulant treatments that increase cytokinin levels under salt stress have been associated with higher isopentenyladenosine content and yield stability in crops such as lettuce (Benito et al., 2024).

Gibberellin-like compounds present in some bacterial CFSs may further support growth under salinity. In plants, gibberellins bind to the GID1 receptor, promoting degradation of DELLA proteins, which are negative regulators of GA signaling. This releases downstream growth-promoting genes, including EXPANSINs and GA20ox, facilitating stem elongation and early seedling development even under osmotic stress (Gupta et al., 2021). Growth promotion observed in CFS-treated seedlings under saline conditions is consistent with partial restoration of GA-dependent growth processes. PGPR-based formulations that elevate GA content in chickpea and soybean under salt stress similarly restore growth while maintaining ionic and redox homeostasis (Basu et al., 2023).

Salinity stress is typically associated with elevated abscisic acid (ABA) accumulation, which suppresses growth to prioritize survival. Several studies indicate that CFS treatments can modulate ABA-related responses, either by attenuating excessive ABA accumulation or by fine-tuning ABA-dependent signaling pathways. This modulation allows plants to maintain essential stress responses, such as stomatal regulation, while alleviating excessive growth inhibition (Khan et al., 2019). For instance, PGPR inoculation often lowers leaf ABA in salt-stressed rice and chickpea while still improving water balance and K^+^/Na^+^ ratios, illustrating the type of hormonal rebalancing that CFSs attempt to mimic (Khan et al., 2021; Asif et al., 2023).

Ethylene production is also enhanced under salinity stress due to increased activity of ACC synthase (ACS) and ACC oxidase (ACO), leading to premature senescence and chlorosis. CFSs derived from ACC deaminase-producing bacteria reduce ethylene levels by degrading the ethylene precursor ACC, thereby alleviating ethylene-induced growth inhibition and leaf senescence under saline conditions (Tyśkiewicz et al., 2022). This ACC deaminase–linked reduction in stress-related ethylene is a recurrent feature of salt-tolerant PGPR, contributing to longer roots, improved water uptake, and better biomass accumulation under high NaCl conditions (Khan et al., 2019; Kumar A. et al., 2020).

In addition to growth hormones, defense-related hormones may also be influenced by CFS metabolites. Salicylic acid (SA) and jasmonic acid (JA) regulate signaling pathways that play important roles in coordinating stress tolerance. Elevated SA levels activate NPR1 and TGA transcription factors, inducing defense-related genes such as PR1 and PR5 (Cao et al., 1994; Després et al., 2003; Zavaliev and Dong, 2024), while JA signaling components, including COI1 and JAZ repressors, regulate stress-responsive genes such as PDF1.2 and LOX2 (Xie et al., 1998; Devoto et al., 2002; Chini et al., 2007; Johnson et al., 2023);. Modulation of these pathways by CFS treatments may contribute to enhanced stress resilience through coordinated growth/defense regulation. Recent studies with halotolerant endophytes show that bacterial inoculation (and by extension their secreted metabolites) can differentially adjust SA, JA and ABA levels in rice under varying salinity levels, supporting a model where CFSs fine-tune defense signaling rather than simply up- or downregulating a single hormone (Kaushal, 2020).

Overall, CFSs support salinity tolerance through coordinated modulation of multiple phytohormone-associated signaling pathways. Rather than acting through a single hormonal route, CFS-derived metabolites appear to rebalance growth- and stress-related hormonal networks, enabling adaptive transcriptional responses that sustain plant growth and physiological stability under saline conditions. However, most mechanistic inferences are still extrapolated from whole-cell PGPR studies; only a limited number of published works combine direct CFS metabolite profiling with hormone measurements *in planta*, underscoring the need for integrated omics to causally link specific CFS components to defined hormonal outcomes (Kumar A. et al., 2020; Roy et al., 2021; Krishnamoorthy et al., 2022).

##### 2.2.1.3 Antioxidant enzyme activation

Salinity stress generates excessive reactive oxygen species (ROS), including superoxide (O_2_⁻), hydrogen peroxide (H_2_O_2_), hydroxyl radicals (·OH), which collectivity trigger oxidative damage in plant cells (Mano, 2012; Foyer and Hanke, 2022). One of the primary consequences of ROS overproduction is lipid peroxidation, a chain reaction initiated by hydrogen abstraction from polyunsaturated fatty acids in membrane lipids. This process leads to the formation of peroxyl radicals, propagation of membrane damage, and accumulation of malondialdehyde (MDA), a well-established biomarker of cellular injury (Mano, 2012; Moldogazieva et al., 2023).

Cell-free supernatants enhance plant antioxidant defenses through two complementary mechanisms: (i) provision of antioxidant or redox-active metabolites that supplement endogenous plant defenses, and (ii) modulation of plant antioxidant responses through ROS-responsive signaling pathways (Hassan et al., 2020; Kibria et al., 2017). In plants, ROS accumulation activates mitogen-activated protein kinase (MAPK) cascades, which can include MPK3 and MPK6, thus regulating stress-responsive transcription factors such as WRKYs, ZATs, and DREBs. These transcription factors control the expression of key antioxidant enzyme genes, including SOD, CAT, APX1, and glutathione peroxidase (GPX). Enhanced antioxidant enzyme activities and, in some cases, increased transcript abundance of these genes have been reported following CFS application under salinity stress, consistent with strengthened ROS detoxification capacity (Kumar A. et al., 2020).

In addition, CFSs may influence redox-sensitive regulatory pathways involving transcription factors such as NPR1 and TGA, particularly in association with (SA) signaling, thereby contributing to coordinated regulation of antioxidant defenses (Després et al., 2003; Pieterse et al., 2009; Herrera-Vásquez et al., 2015; Jamil et al., 2022). Bacterial metabolites present in CFSs, including lipopeptides and volatile organic compounds (VOCs), can function as elicitors that activate antioxidant machinery independent of microbial colonization. These metabolites contribute to maintaining cellular redox homeostasis by enhancing the ascorbate-glutathione cycle, as reflected by increased GSH: GSSG ratios and elevated activities of glutathione reductase and dehydroascorbate reductase (Vaish et al., 2020).

Lipid peroxidation proceeds through a radical chain reaction consisting of three phases: initiation, propagation, and termination. During initiation, hydroxyl radicals (·OH) extract hydrogen atoms from membrane lipids; during propagation, lipid radicals react with oxygen to form peroxyl radicals that attack adjacent lipids; and termination results in membrane structural breakdown. This cascade generates toxic aldehydes such as acrolein, 4-hydroxynonenal (HNE), and benzaldehyde, which further amplify cellular damage (Nurbekova et al., 2021; Nurbekova et al., 2024). CFSs mitigate this process through both non-enzymatic and enzymatic mechanisms. Non-enzymatic components, such as exopolysaccharides and osmoprotectants (e.g., proline), can directly scavenge ROS and limit initiation, while enzymatic pathways, via upregulation of SOD, CAT, and APX, interrupt propagation and reduce peroxide accumulation (Sharma et al., 2016; Kumar et al., 2020). Recent studies further support the role of microbial metabolites in suppressing lipid peroxidation. Zholdasbek et al. (2026) reported that metabolite-rich bacterial extracts reduced MDA accumulation by up to 45% in salt−stressed wheat, consistent with enhanced SOD, CAT, and APX activities. Likewise, Rakhmatova et al. (2026) demonstrated that metabolite fractions stabilized membrane lipids and limited ROS propagation in barley, providing additional evidence that microbial metabolites can directly modulate oxidative damage pathways.

Empirical evidence from crops such as soybean indicates that CFS treatment under saline conditions significantly reduces superoxide accumulation and malondialdehyde (MDA) content, both reliable indicators of oxidative damage, while simultaneously enhancing enzymatic ROS-scavenging activities. This reduction in oxidative stress contributes to the preservation of chlorophyll content, thylakoid membrane integrity, and overall photosynthetic stability under salt stress (Kumar A. et al., 2020). Collectively, these findings support antioxidant defense activation as a central mechanism by which CFSs enhance plant cellular viability and redox equilibrium during exposure to salinity stress.

However, most studies remain limited to controlled greenhouse conditions, with relatively few field-scale validations. Additionally, the relative contribution of direct antioxidant metabolites versus host-mediated transcriptional regulation remains insufficiently quantified.

##### 2.2.1.4 Ion homeostasis and transport

A major mechanism through which CFSs alleviate salinity stress involves the regulation of ion homeostasis, a core determinant of plant survival under high−salt conditions. Salinity stress induces ionic toxicity primarily through excessive accumulation of sodium ions (Na^+^) in the plant cytoplasm, which disrupts enzyme activities, impairs nutrient uptake, particularly potassium (K^+^), and destabilizes membrane potentials. To counteract these effects, plants employ selective Na^+^ exclusion, vacuolar sequestration, and intracellular ion redistribution, processes regulated by stress−activated signaling networks.

A central mechanism underpinning salt tolerance is the Salt Overly Sensitive (SOS) pathway. Salt−induced osmotic shock elevates cytosolic Ca²^+^ levels, which are sensed by the calcium−binding protein SOS3. SOS3 forms a complex with the protein kinase SOS2, leading to activation of SOS1, a plasma−membrane Na^+^/H^+^ antiporter that facilitates Na^+^ extrusion and reduces ion toxicity (Zhu, 2003). Emerging evidence suggests that bacterial CFSs can enhance SOS pathway activity under salinity stress as increased SOS1 transcript abundance has been reported in CFS−treated plants, consistent with improved Na^+^ efflux and reduced cytosolic Na^+^ accumulation (Xie et al., 2022).

In parallel, vacuolar sequestration of Na^+^ is mediated by tonoplast−localized Na^+^/H^+^ antiporters such as NHX1, which transport Na^+^ into vacuoles to detoxify the cytoplasm. Several studies report that CFS treatments are associated with increased NHX1 expression and enhanced vacuolar Na^+^ compartmentalization in crops such as soybean and maize; however, comparable evidence in *Arabidopsis thaliana* remains limited, highlighting an important knowledge gap in generalizing CFS−mediated ion transport mechanisms across plant taxa (Xie et al., 2022).

Maintenance of cytosolic K^+^ concentration is equally critical for sustaining enzymatic activity, osmotic balance, and overall cellular function under salinity stress. High−affinity K^+^ transporters (e.g., HAK5) and inward−rectifying K^+^ channels (e.g., AKT1) are regulated by Ca²^+^−dependent signaling modules involving calcineurin B−like (CBL) proteins and CBL−interacting protein kinases (CIPKs). The CBL1–CIPK23 complex phosphorylates AKT1, thereby enhancing K^+^ uptake during salt stress (Chen et al., 2007; 2024; Yao et al., 2010). While CFS treatments may influence this regulatory network indirectly through modulation of upstream calcium signaling or elicitor−like microbial metabolites, direct evidence linking CFS application to activation of the CBL–CIPK–AKT1 module is currently lacking.

At the physiological level, multiple studies in soybean and maize demonstrate that CFS application under salinity stress substantially reduces tissue Na^+^ accumulation (by approximately 2–4.5−fold) while increasing K^+^ content (by about 9–67%), as reported for soybean and maize (Xie et al., 2022). These shifts markedly improve the cytoplasmic K^+^/Na^+^ ratio, a key determinant of salt tolerance and metabolic stability (Kumar A. et al., 2020; Xie et al., 2022). CFS treatments may also contribute to improved chloride (Cl⁻) management, potentially through effects on anion transport or enhancement of root barrier properties; however, direct mechanistic evidence for CFS−mediated Cl⁻ exclusion remains limited.

Through coordinated effects on ion transporter expression, membrane stability, and ion partitioning, CFS−treated plants maintain selective ion uptake and preserve essential physiological processes, including protein synthesis, photosynthesis, and turgor regulation. Collectively, these responses underscore ion homeostasis as a central mechanism by which CFSs enhance salinity tolerance in crops, while also emphasizing the need for further mechanistic validation across diverse plant species and experimental systems. This suggests that CFSs contribute to salinity tolerance through integrated regulation of ion transport systems rather than isolated modulation of individual transporters.

##### 2.2.1.5 Osmotic adjustment and compatible solutes

Salinity stress induces osmotic imbalance in plants by lowering soil water potential, thereby restricting water uptake by roots and reducing cellular turgor. To maintain osmotic homeostasis under these conditions, plants accumulate compatible solutes (osmoprotectants) such as proline, glycine betaine, trehalose, and soluble sugars. These low-molecular-weight compounds contribute to cellular hydration, stabilize proteins and membranes, and protect cellular structures from stress-induced damage (Turner, 2018).

Cell-free supernatants can enhance plant osmotic adjustment by influencing the accumulation of compatible solutes and, in some cases, by modulating expression of genes involved in their biosynthesis. Proline accumulation is among the most consistently reported responses associated with CFSs treatment under salinity stress. Increased expression of P5CS (Δ¹-pyrroline-5-carboxylate synthetase), a key enzyme in proline biosynthesis, has been observed in plants exposed to microbial metabolites, supporting enhanced proline production under stress conditions (Sharma et al., 2016). Proline functions as an effective osmo-protectant by maintaining cellular hydration, stabilizing proteins and membranes, and scavenging reactive oxygen species (ROS), thereby linking osmotic adjustment with oxidative stress mitigation (Fatima et al., 2020).

Trehalose biosynthesis involves the coordinated action of trehalose-6-phosphate synthase (TPS) and trehalose-6-phosphate phosphatase (TPP). Trehalose and its intermediate trehalose-6-phosphate (T6P) play dual roles as osmoprotectants and signaling molecules, contributing to membrane stabilization, protection of macromolecules, enhancement of antioxidant defenses, and coordination of carbon allocation under stress (Paul et al., 2008; Mollavali and Börnke, 2022). While direct evidence for CFS-induced activation of trehalose biosynthesis pathways remains limited, the physiological relevance of trehalose-mediated osmotic protection under salinity is well established, and emerging studies suggest that microbial metabolites may influence this pathway (Mollavali and Börnke, 2022).

Glycine betaine represents another important compatible solute that stabilizes photosynthetic complexes and enzyme systems, allowing essential metabolic processes to continue under saline conditions. CFSs treatments may contribute to increased glycine betaine accumulation by supplying metabolic precursors or by influencing expression of biosynthetic genes such as BADH (betaine aldehyde dehydrogenase). Enhanced glycine betaine levels have been associated with improved photosynthetic efficiency and strengthened antioxidant defenses in salt-stressed plants (Giri, 2011; Xu et al., 2018; Khalid et al., 2022).

In addition to nitrogen-containing osmolytes, CFSs treatments are frequently associated with increased accumulation of soluble sugars. These sugars contribute to osmotic balance, stabilize cellular structures, and provide readily available energy to sustain metabolism during the early phases of salinity stress. In several studies, CFS application has been linked to altered sugar metabolism and transport, consistent with improved turgor maintenance and delayed stress-induced growth inhibition (Fatima et al., 2020; Kumar et al., 2020). Key compatible solutes, their biosynthetic pathways, and reported modulation by CFSs are summarized in **Table 3**.

**Table 3:** Compatible solute metabolism and modulation by bacterial CFSs under salinity stress.

Compatible solute	Biosynthetic genes/enzymes	Roles in salinity stress	Influence associated with CFS application	References
Proline	P5CS (Δ¹-pyrroline-5-carboxylate synthetase)	Osmotic balance, protein stabilization, ROS scavenging	Increased proline accumulation: in some studies, associated with elevated P5CS expression	Sharma et al., 2016
Trehalose	TPS, TPP	Membrane/protein stabilization, antioxidant defense, stress signaling	Limited but emerging evidence suggesting possible stimulation of trehalose-related pathways	Ponnu et al., 2011
Glycine Betaine	BADH	Photosystem protection, enzyme stabilization, osmoprotection	May enhance glycine betaine accumulation via precursor supply or modulation of BADH expression	Giri, 2011; Khalid et al., 2022; Xu et al., 2018.
Soluble sugars	Invertases, sucrose synthases, sugar transporters	Maintain cell turgor, stabilize structures, energy source	Increased soluble sugar levels; associated with altered sugar metabolism and transport	Fatima et al., 2020; Kumar et al., 2020.

These responses highlight that osmotic regulation by CFSs operates in synergy with antioxidant and ionic balance mechanisms.

Collectively, by promoting the accumulation of compatible solutes, CFSs support plant water retention, protect vital cellular components, and sustain metabolic activity during salinity stress. This osmotic adjustment represents a critical early defense mechanism that complements hormonal regulation, antioxidant activation, and ion homeostasis in CFS-mediated salinity tolerance.

##### 2.2.1.6 Nutrient mobilization and rhizosphere effects

Beyond hormonal regulation, redox balance, and ion homeostasis, CFSs contribute to salinity stress tolerance by improving nutrient availability and influencing rhizosphere processes. Salinity commonly restricts nutrient acquisition, particularly phosphorus (P), iron (Fe), and essential micronutrients, due to elevated soil pH, ionic antagonism, reduced nutrient solubility, and impaired root growth (Shrivastava and Kumar, 2015; Polash et al., 2019). Cell-free supernatants can partially alleviate these constraints by delivering metabolite-based nutrient solubilizers, chelators, and signaling molecules that facilitate nutrient mobilization and uptake (Alori et al., 2025). Phosphorus mobilization represents a major benefit, as phosphate frequently precipitates as insoluble complexes with calcium, iron, or aluminum in saline soils (Singh et al., 2022c). Cell-free supernatants derived from phosphate-solubilizing bacteria contain organic acids such as gluconic, citric, and oxalic acids, which chelate cations and release bioavailable phosphate (Mosela et al., 2022; Pan and Cai, 2023). In addition, some CFSs may include phosphatases and phytases capable of mineralizing organic phosphorus pools, thereby enhancing plant phosphorus acquisition under salt stress (Mosela et al., 2022).

Iron mobilization is similarly supported through microbial siderophores, including high-affinity Fe³^+^ chelators such as pyoverdine and bacillibactin, which have been identified in CFSs from several beneficial bacterial strains (Hoffmann et al., 2002; Cornelis et al., 2023). These siderophores scavenge iron from insoluble soil fractions, increasing iron availability either directly or indirectly through changes in rhizosphere chemistry and root exudation (Singh et al., 2022a). Plants respond to improved iron availability by activating iron-regulated transport systems such as IRT1, facilitating iron uptake even under salt-induced nutrient limitation (Mehmood et al., 2023).

In addition to direct nutrient mobilization, CFSs can influence rhizosphere dynamics through the activity of microbial metabolites, including quorum-sensing molecules, volatile organic compounds (VOCs), and biosurfactants. These compounds may stimulate indigenous beneficial microorganisms, modify root exudation patterns, and improve root–soil interactions, collectively enhancing microbial activity and nutrient-use efficiency. CFS-associated rhizosphere acidification has also been linked to increased solubility of micronutrients such as zinc (Zn), copper (Cu), and manganese (Mn), which are essential for chlorophyll biosynthesis, redox metabolism, and enzyme function (Shrivastava and Kumar, 2015; Polash et al., 2019).

Although often underemphasized, nutrient mobilization plays a critical role in sustaining plant metabolism when salinity impairs root function. By increasing the availability of phosphorus, iron, and micronutrients, CFSs indirectly support photosynthesis, protein synthesis, and energy metabolism under saline conditions. This nutrient-driven reinforcement acts synergistically with hormonal regulation, antioxidant activation, and osmotic adjustment, providing a comprehensive physiological advantage for plant survival and performance in salt-affected soils (Vacheron et al., 2013; Qin et al., 2016).

Building upon these mechanistic insights into how *Bacillus* and *Pseudomonas* CFSs influence plant nutrient dynamics and rhizosphere processes, the following section examines how these metabolite-mediated effects translate into practical crop improvement strategies under real-world agricultural conditions.

#### 2.2.2 Cell-free supernatants and salinity stress alleviation (application)

Cell-free supernatants derived from *Bacillus* and *Pseudomonas* strains have been widely evaluated under NaCl-induced salinity stress in cereals, vegetables, and salt-sensitive oilseeds such as soybean and canola. Across controlled-environments and pot experiments, CFS application consistently improves early-stage performance, including seed germination, seedling vigor, root elongation, and shoot biomass, under salinity stress. These improvements are frequently accompanied by enhanced chlorophyll content reduced electrolyte leakage, and better maintenance of K^+^/Na^+^ homeostasis, indicating protection of photosynthetic and membrane stability processes (Shah et al., 2022; Xie et al., 2022). Beyond salinity, several studies report that CFSs mitigate combined or sequential stresses, such as salinity with drought or heavy metals, suggesting that metabolite-rich formulations may confer broad-spectrum abiotic stress tolerance (El-Gendi et al., 2022; Trinh et al., 2025). Although most evidence comes from pre−agronomic and greenhouse studies, these findings highlight the potential of CFS−based treatments as practical tools for salinity stress mitigation, while emphasizing the need for standardized field−level validation (Dhar et al., 2024).

*Bacillus*-derived CFSs have been most widely tested, reflecting the ease of cultivating *Bacillus* spp. at scale and their ability to secrete diverse metabolites such as phytohormones, osmoprotectants, lipopeptides, and antioxidant compounds. In cereals, vegetables and several oilseed crops, CFSs from halotolerant *Bacillus* strains have been shown to enhance germination and early seedling growth at NaCl levels ranging from 50–200 mM NaCl, often through increased chlorophyll content, reduced electrolyte leakage, and improved membrane stability. Multiple studies further demonstrate that *Bacillus*−derived CFSs promote Na^+^ exclusion and K^+^ retention in roots and shoots, thereby maintaining a favorable K^+^/Na^+^ ratio and supporting photosynthetic efficiency under salinity stress (Peng et al., 2023; Pérez-Inocencio et al., 2023).

Although *Pseudomonas* CFSs have been evaluated less extensively than those from *Bacillus*, available studies indicate comparable efficacy under salinity stress because *Pseudomonas* spp. secrete high levels of siderophores that enhance Fe acquisition (Ahmed and Holmström, 2014; Gao et al., 2022), phytohormones such as IAA and cytokinins that stimulate root system architecture (Egamberdieva et al., 2017; Xie et al., 2022), organic acids that improve nutrient solubilization (Glick, 2014; Khan et al., 2016), and antioxidant metabolites that mitigate ROS accumulation (Peng et al., 2023; Pérez-Inocencio et al., 2023) Across multiple crops, *Pseudomonas*−derived CFSs stimulate root elongation, increase root surface area, and enhance water−use efficiency, while simultaneously improving the uptake of phosphorus, iron, and essential micronutrients. These physiological improvements frequently translate into increased shoot biomass and, in some cases, enhanced reproductive traits; however, yield−level evidence remains limited, particularly for field−grown oilseeds where CFS−specific trials are still scarce. A few studies, also report that *Pseudomonas*−derived CFSs mitigate combined salinity and heavy−metal stress, suggesting broader applicability in multi−stress soils where ionic toxicity and osmotic imbalance co−occur (Gao et al., 2022; Peng et al., 2023; Pérez-Inocencio et al., 2023).

Application mode critically influences the efficiency of *Bacillus* and *Pseudomonas* CFSs in mitigating salinity stress. Seed priming is the most common approach and involves soaking seeds in diluted CFS for 6–24 h, depending on crop species, allowing uptake of bioactive metabolites before sowing. This enhances germination rate, seedling uniformity, and early root development, equipping seedlings to better tolerate the initial osmotic shock imposed by saline soils (Adhikari et al., 2020). Root dipping during transplanting exposes young roots to concentrated CFS metabolites, improving early ion homeostasis and nutrient mobilization during establishment (Alori et al., 2025). Soil drenching delivers CFS directly to the rhizosphere, supporting sustained metabolite availability during vegetative growth and enhancing K^+^/Na^+^ regulation under salinity (Gao et al., 2022). Foliar application, although less common, targets photosynthetic tissues and primarily enhances antioxidant defenses and chlorophyll stability under salt−induced oxidative stress (Sánchez et al., 2023). Despite these promising outcomes, comparative evaluations of application modes, particularly under field conditions and across diverse crops such as soybean, canola, and wheat, remain scarce and represent a critical knowledge gap limiting large−scale deployment (Abulfaraj and Jalal, 2021).

Wheat has been a focal crop for validating these application strategies under saline conditions. For example, Vimal et al. (2018) demonstrated that salt-tolerant *Bacillus* and *Pseudomonas* strains improved wheat growth and yield using rhizosphere and seed treatments, highlighting the practical viability of these genera. Oilseed crops, including soybean and canola, exhibit considerable salt sensitivity, yet few reports evaluating CFSs specifically. Most existing literature pertains to live PGPB inoculants, which have shown promising effects on growth and yield under salt stress but face challenges in consistency and shelf life. In contrast, studies focusing specifically on CFS-driven improvements in grain yield, oil content, and seed quality in soybean and canola under field-relevant salinity are still rare, underscoring the need for dedicated trials that quantify physiological responses (e.g., ion homeostasis, antioxidant defense) alongside agronomic outputs of these economically important crops (Abulfaraj and Jalal, 2021). Bridging this gap through standardized, multi-location field research will be key to translating CFS technology into promising, climate-resilient solutions for salt-affected oilseed production.

The practical utility of these application modes is particularly significant in high-stress agroecosystems, such as the Canadian Prairies, where soil salinity often coincides with fluctuating summer temperatures and erratic precipitation. In these regions, CFS application, especially through seed priming, serves as a strategic metabolic priming tool. By bolstering early−stage physiological robustness, CFS treatments can equip seedlings to navigate simultaneous osmotic and heat stress, providing a regionally relevant solution for stabilizing yields in climate−vulnerable agricultural zones (Goverment of Canada, 2024). Despite promising application outcomes and regional relevance, challenges such as formulation variability, limited field validations, and regulatory uncertainties must be addressed to fully realize the potential of *Bacillus* and *Pseudomonas* CFSs as sustainable solutions for salinity stress management.

#### 2.2.3 Challenges in the use of *Bacillus* and *Pseudomonas* CFSs

Despite promising results, several challenges impede the broader adoption and efficacy of *Bacillus* and *Pseudomonas* CFSs in agricultural salinity management. Given that the ability of CFSs to enhance plant growth is tied to bioactive metabolites in the supernatant, this comes with several challenges, especially since details on the quantity and quality of such metabolites are often unclear. Below are some of the main challenges associated with using CFSs to enhance plant growth under saline conditions.

##### 2.2.3.1 Absence of uniform standard controls across researchers

Beyond the presence of bioactive metabolites, the reliability of CFS bioactivity also depends on appropriate experimental design. To ensure that observed plant responses to CFS are attributable to bioactive microbial metabolites rather than residual media components or processing artifacts, the inclusion of appropriate experimental controls is essential in CFS studies. A rigorous assessment of CFS-mediated bioactivity requires the inclusion of stringent experimental controls to decouple microbial metabolite effects from confounding variables, such as residual media constituents, pH fluctuations, and osmotic artifacts. Key experimental controls include (i) uninoculated media controls (processed identically via centrifugation and filtration) to account for baseline nutrient-driven growth; (ii) pH-stabilized CFS treatments to isolate metabolite-specific signaling from pH-induced physiological shifts; (iii) heat-inactivated controls (e.g., autoclaved CFS) to differentiate between thermostable metabolites and residual enzymatic activity; and (iv) sterile filtrates to ensure results are not skewed by processing-related artifacts. The absence of such controls may lead to misinterpretation of CFS bioactivity, particularly when residual nutrients, salts, or pH differences independently influence plant responses. This in turn may slow the introduction of CFSs into the biostimulant market and even when they are, inconsistencies may be inevitable. **Table 4** summarizes the extent to which these controls have been implemented across the current literature. Future research should prioritize standardized experimental designs and explicit reporting of control strategies to strengthen causal inference and reproducibility.

**Table 4:** Evaluative summary of experimental controls in CFS-salinity. .

Control category	Functional rationale	Studies utilizing control	Studies lacking control
Uninoculated Media	Distinguishes microbial metabolites from baseline medium nutrition.	Naamala et al., 2022; Subramanian et al., 2021; Fan and Smith, 2022	Adhikari et al., 2020; Kumar et al., 2020; Shah et al., 2022
pH-Stabilized Media	Accounts for growth effects induced by medium acidification/alkalization.	Abdelkefi et al., 2024; Trinh et al., 2025	Majority of reviewed literature
Heat-Inactivated CFS	Eliminates enzymatic activity while retaining thermostable metabolites.	Wang et al., 2022	Majority of reviewed literature
Sterile Filtration	Controls for artifacts introduced during mechanical sterilization.	Subramanian et al., 2013	Majority of reviewed literature
Comprehensive (≥3 Types)	Ensures robust mechanistic attribution to microbial bioactive.	7 Identified Studies	19 Identified Studies (73%)

As demonstrated in **Table 3**, the absence of comprehensive controls represents a critical methodological gap in current CFS research. Without these rigorous benchmarks, observed improvements in plant growth or salt-stress resilience may inadvertently reflect basal medium nutrition, pH modulation, or osmotic artifacts rather than the activity of specific microbial metabolites. To move the field from largely putative observations to more firmly established mechanisms, future CFS studies must prioritize standardized control frameworks. Such methodological rigor is indispensable for accurate mechanistic interpretation and the successful translation of CFS technologies from controlled environments to field-scale applications. Developing community-endorsed minimum reporting standards for CFS preparation, characterization, and controls would further support reproducibility and facilitate translation of CFS-based technologies to field-scale applications.

##### 2.2.3.2 Variability in composition of bioactive metabolites

The quantity and quality of bioactive metabolites in CFSs varies significantly depending on factors such as microbial species, growth medium composition and growth environment conditions (Qadi et al., 2023; Ahsan et al., 2026). Moreover, in most cases the quality and quantity of active metabolites in CFS remains largely unknown before use. Even after analysis with tools like mass spectrometry, novel metabolites remain unidentified, requiring long processes to do so. This presents several key limitations that hinder their reliability and application. Without precise knowledge of metabolite concentrations, it is difficult to control dosage, leading to inconsistent plant responses that may range from ineffective to potentially inhibitory (Naamala et al., 2022; Msimbira et al., 2022). Variability in bacterial growth conditions further contributes to batch-to-batch differences in composition, reducing reproducibility across experiments and field applications. Additionally, the lack of defined active compounds limits mechanistic understanding, making it challenging to link specific metabolites to observed plant responses. This uncertainty also complicates standardization, scalability, and regulatory approval, as consistent formulation and safety profiles cannot be guaranteed (Alori et al., 2025). Furthermore, complex mixtures may contain antagonistic compounds that negatively affect efficacy. Overall, the absence of quantitative and compositional clarity in CFS restricts their scientific validation, practical deployment, and commercial development. **Table 5** synthesizes application parameters-concentration ranges, dilution factors, delivery methods, and timing-alongside physiological outcomes reported by different researchers. Seed priming protocols typically use 5-20% (v/v) CFS, root dipping higher concentrations (10-25% v/v), and soil drenching lower ranges (3-8% v/v). Foliar sprays often employ intermediate levels (10-15% v/v). Even with these percentages stated, it is difficult to determine the real percentage of the bioactive compounds, especially those that are bringing about the biostimulation. Application timing spans from germination (improving emergence and early vigor) to vegetative and tillering stages (mitigating ongoing abiotic stress). These patterns indicate that optimal CFS efficacy is tightly linked to crop−specific salinity thresholds and developmental stage.

**Table 5:** Summary of CFS application parameters, concentration ranges, and salinity alleviation outcomes across different crop species and growth stages.

Study	Crop	Salinity level	Application method	Concentration/dilution	Growth stage	Key outcomes
Naamala et al., 2022	Soybean	100 mM NaCl	Seed priming	10% CFS (v/v)	Germination	+35% germination, +28% radicle length
Fan and Smith, 2022	Tomato	100 mM NaCl	Root dipping	20% CFS	Seedling	+42% biomass, chlorophyll retention
Adhikari et al., 2020	Soybean	150 mM NaCl	Soil drench	5% CFS (1:20)	Vegetative	-70% Na^+^ influx, +GA signaling
Shah et al., 2022	Soybean	120 mM NaCl	Foliar spray	15% CFS	Vegetative	SOD/CAT +45%, MDA -32%
Subramanian et al., 2021	Canola	80 mM NaCl	Seed priming	5-10% CFS	Germination	Root elongation +30%, nutrient uptake
Wang et al., 2022	Wheat	200 mM NaCl	Soil drench	3% CFS (1:33)	Vegetative	Yield +22%, ion homeostasis
Shah et al., 2022	Maize	100 mM NaCl	Root dipping	25% CFS	Seedling	Germination +29%, FW/DW +18%
Trinh et al., 2025	Rice	120 mM NaCl	Foliar + soil	10% CFS	Tillering	ROS -40%, proline +52%
Xie et al., 2022	Maize	150 mM NaCl	Soil drench	8% CFS	Vegetative	SOS1/NHX1 ↑, Na^+^ -3.2-fold
Hashem et al., 2019	Wheat	90 mM NaCl	Seed treatment	15% CFS	Germination	Germination +31%, biomass +25%

A comparative analysis of **Table 4** indicates that CFS efficacy is strongly influenced by both application method and concentration. Notably, optimal ranges vary across crops and salinity levels, highlighting the absence of a universal application strategy. For example, wheat responses under severe salinity (200 mM NaCl) differ markedly from those observed in soybean under moderate stress (100–150 mM NaCl), indicating that CFS performance is highly context dependent. Consequently, CFSs should be considered dynamic formulations whose effectiveness depends on microbial origin, metabolite composition, and application strategy. Taken together, the patterns in **Table 4** underscore the need for future studies to report CFS composition, application frequency, and crop developmental stage in a standardized manner, enabling cross−study comparisons and the development of agronomically relevant dosage guidelines.

##### 2.2.3.3 Instability of some bioactive compounds and need for repetitive applications

The stability of CFS metabolites varies, some more than others (e, especially under field conditions, where they are exposed to factors such as UV light, a wide range of pH, extreme temperature and high levels of salt (Kumar R. et al., 2020; Fincheira and Quiroz, 2021). Some metabolites could have a short shelf life, thus degrading during storage or shortly after application, hence, affecting performance (Morcillo et al., 2022; Khan et al., 2023). While numerous studies demonstrate the beneficial effects of CFSs under controlled conditions, including *in vitro* assays, growth chambers, and greenhouse experiments, relatively few have evaluated their performance under field conditions. As a result, much of the current evidence should be considered proof-of-concept rather than field-validated. Differences in soil complexity, microbial competition, environmental variability, and application logistics may significantly influence CFS efficacy under real agricultural settings. However, potential non-target effects of CFS applications on native soil microbiota, beneficial rhizosphere organisms, and broader soil ecological functions remain poorly characterized and should be evaluated in future field-based risk assessments. Therefore, caution is warranted when extrapolating laboratory findings to large-scale deployment, where weather and soil conditions are not controlled. Unlike live microbial cells that could establish meaningful populations in the rhizosphere thereby eliminating or lowering the rate of reapplying, CFS must be applied every season, sometimes more than once in a single growing season, to optimize efficacy. This could discourage crop producers from adopting the technology due to added costs and operational complexity (Chivenge et al., 2011; Khan et al., 2023).

##### 2.2.3.4 Limited field validation

There is limited research on the performance of CFSs under field conditions, and most research to date has been confined to controlled environment or pot experiments. There is a notable lack of multi-location, multi-season field trials assessing CFSs efficacy under diverse soil salinity levels, environmental conditions, and different crop species especially for key crops like soybean and canola (Fan and Smith, 2022). Research has shown that the efficacy of CFS is affected by several factors such as crop species and soil conditions (Msimbira et al., 2022; Naamala et al., 2022). There is need for research on the performance of CFSs across several crop species and environmental conditions.

##### 2.2.3.5 Regulatory and safety concerns

CFSs products fall into ambiguous regulatory categories between biostimulants and biofertilizers, causing uncertainty in registration processes. Safety evaluations require detailed knowledge of residual metabolites, environmental fate, and human and ecosystem health impacts, which are challenging due to chemical complexity and variability across production batches and the need to have all of them studied (Kaushal et al., 2023). Additionally, as mentioned earlier, constituents could possibly change with changing growth conditions, even for a single bacterial strain.

##### 2.2.3.6 Mechanistic gaps

Despite insights into hormonal signaling, antioxidant modulation, and ion transport, integrated, multi−omics analyzes and robust molecular biomarkers that can reliably predict CFS efficacy across crops and environments remain scarce. Enhanced mechanistic understanding would not only facilitate rational design of optimized formulations and targeted applications but also support regulatory approval and product positioning in increasingly evidence−driven biostimulant markets (Santoyo et al., 2024). While CFSs are often viewed as more environmentally stable alternatives to live microbial inoculants, their environmental fate under intensive agricultural use remains a critical knowledge gap. Key bioactive constituents, such as bacteriocins and lipopeptides, exhibit varying degrees of persistence within the soil matrix (Khan et al., 2023). Repeated applications could potentially lead to the accumulation of these compounds, inadvertently disrupting the indigenous rhizosphere microbiota or exerting selective pressure on non−target soil organisms (Morcillo et al., 2022). Moreover, interactions between accumulated CFS metabolites and other agrochemicals (e.g., fertilizers, pesticides) are largely unexplored, further complicating predictions of long−term rhizosphere responses. To transition toward sustainable deployment, future research must prioritize evaluating the long−term ecological footprint of CFS applications, specifically focusing on the degradation rates of microbial bioactive compounds and their interactions with native soil community structures to ensure ecosystem health is not compromised.

Thus, in addition to the formulation, regulatory, and mechanistic challenges outlined above, thoroughly characterizing the long−term environmental footprint and rhizosphere consequences of CFS use remains a key barrier to their safe, large−scale deployment.

## 3 Conclusions and future directions

Bacterial CFS derived from *Bacillus* and *Pseudomonas* species represent promising biostimulant formulations capable of alleviating salinity stress in key crops such as soybean, canola, and wheat. These metabolite-rich preparations consistently enhance seed germination, seedling vigor, biomass accumulation, photosynthetic efficiency, and ionic homeostasis under salt-imposed osmotic challenges. The mechanistic basis of these benefits encompasses modulation of hormonal signaling, antioxidant defense, ion transport regulation, and improved nutrient acquisition, aligning with the broader functions of live PGPB but offering advantages in formulation stability and regulatory acceptance (Sharma et al., 2016).

While *Bacillus* and *Pseudomonas* CFSs have demonstrated clear bioactivity in plant systems, their large-scale practical deployment remains constrained by challenges such as batch-to-batch variability, insufficient multi-environment field validations, limited knowledge regarding optimal application protocols, regulatory uncertainties, and incomplete mechanistic understanding at molecular and biochemical levels. Overcoming these barriers demands multi-location, multi-season field trials with standard quality control benchmarks, advanced omics-guided screening to identify key bioactive metabolites and their modes of action, and development of durable, user-friendly formulations compatible with current crop production systems (Santoyo et al., 2024). A paradigm shift from complex supernatant mixtures toward defined, purified, or semi−purified microbial−derived metabolites (MDMs) with known structures, mechanisms, and dose–response relationships is necessary. This transition would address key limitations of crude CFSs, including batch−to−batch variability, unclear modes of action, inconsistent metabolite concentrations, and challenges in standardization, reproducibility, and regulatory acceptance. Importantly, many of the bioactive compounds isolated from CFSs are microbe−to−plant signal molecules-such as lipo−chitooligosaccharides (LCO) and the bacteriocin thuricin 17 (Subramanian and Smith, 2015; Gray et al., 2006) that plants perceive through dedicated signaling pathways. Purifying CFSs to obtain these individual signaling compounds enables formulation strategies that enhance stability, reduce wastage, and facilitate intellectual property protection. Advances in analytical chemistry and metabolomics now make it feasible to isolate and characterize such compounds (Wang et al., 2016; Ilangumaran and Smith, 2017; Kaushal et al., 2023). Furthermore, research on pure compounds has demonstrated their ability to enhance plant growth across a wide range of crop species, at minute concentrations, under both stressed and unstressed conditions. For example, the bacteriocin thuricin 17 (recently renamed Bacillin 20), produced by *Bacillus thuringiensis* NEB17, enhances soybean germination under 100 mM NaCl stress (Subramanian et al., 2016), and promotes root and shoot growth and biomass accumulation in several crops, including canola, soybean, and switchgrass (Subramanian et al., 2021; Nazari and Smith, 2023; Eisvand and Smith, 2025).

Future research should prioritize crop- and region-specific adaptations, particularly for salt-sensitive oilseeds in agroecologically complex zones such as the Canadian Prairies, where combined salinity, drought, and heat stresses challenge productivity. Innovations in seed priming technology utilizing CFSs, combined with complementary agronomic practices, hold the potential to improve stress resilience synergistically. Clear regulatory frameworks and economic viability assessments will be indispensable in allowing transition CFSs from promising experimental products to widely adopted solutions supporting climate-change-resilient agriculture.

In conclusion, unlocking the full agronomic potential of *Bacillus* and *Pseudomonas* CFSs for salinity stress alleviation demands coordinated interdisciplinary efforts spanning microbiology, biochemistry and agronomy, combined with regulatory considerations. Overall, advancing CFS-based strategies will require coordinated efforts integrating microbiology, plant physiology, and agronomy to enable their reliable application in climate-resilient agricultural systems.
